# Organic molecular heterogeneities can withstand diagenesis

**DOI:** 10.1038/s41598-017-01612-8

**Published:** 2017-05-04

**Authors:** Julien Alleon, Sylvain Bernard, Corentin Le Guillou, Damien Daval, Feriel Skouri-Panet, Maïa Kuga, François Robert

**Affiliations:** 1 Institut de Minéralogie, de Physique des Matériaux et de Cosmochimie (IMPMC), Sorbonne Universités - CNRS UMR 7590, Muséum National d’Histoire Naturelle, UPMC Univ. Paris 06, IRD UMR 206, 61 rue Buffon, 75005 Paris, France; 2 UMET, CNRS UMR 8207, Université Lille 1, 59655 Villeneuve d’Ascq, France; 3 Laboratoire d’Hydrologie et de Géochimie de Strasbourg, Université de Strasbourg/EOST - CNRS UMR 7517, 1 Rue Blessig, 67084 Strasbourg, France; 4 Department of Earth Sciences, ETH Zürich, 8092 Zürich, Switzerland; 5 Department of Earth Atmospheric and Planetary Sciences, Massachusetts Institute of Technology, Cambridge, MA 02139 USA

## Abstract

Reconstructing the original biogeochemistry of organic fossils requires quantifying the extent of the chemical transformations that they underwent during burial-induced maturation processes. Here, we performed laboratory experiments on chemically different organic materials in order to simulate the thermal maturation processes that occur during diagenesis. Starting organic materials were microorganisms and organic aerosols. Scanning transmission X-ray microscopy (STXM) was used to collect X-ray absorption near edge spectroscopy (XANES) data of the organic residues. Results indicate that even after having been submitted to 250 °C and 250 bars for 100 days, the molecular signatures of microorganisms and aerosols remain different in terms of nitrogen-to-carbon atomic ratio and carbon and nitrogen speciation. These observations suggest that burial-induced thermal degradation processes may not completely obliterate the chemical and molecular signatures of organic molecules. In other words, the present study suggests that organic molecular heterogeneities can withstand diagenesis and be recognized in the fossil record.

## Introduction

The fossil record contains crucial information about the evolution of Life on Earth^1, 2^. Molecular investigations regarding ancient Life are yet limited by the poor quality of the ‘biogeochemical’ signals preserved in the fossil record: in addition to biodegradation, burial-induced alteration processes (i.e. thermal maturation) inevitably modify the original biochemical signatures of fossilized organic molecules^1, 3^. With increasing temperature, weaker organic bonds are thermally broken and significant deoxygenation and dehydrogenation reactions occur^1, 4^. Thermal degradation eventually promotes increasing structural reorganization and may ultimately lead to the formation of pure graphite^5, 6^.

It has long been recognized that biomacromolecules exhibit conspicuous differences in decay in natural environments^7, 8^. For instance, cell-wall biopolymers that protect some algae, cysts, spores and pollen grains are intrinsically more resistant than polysaccharides, proteins and nucleic acids^9–11^. In some contexts, taxon-specific chemosystematic data can even be preserved in the fossil record^12, 13^. Still, the general perception in paleobiology remains that thermal maturation processes lead to a converging composition of organic materials from different origins, thereby limiting the use of chemical composition for discriminating between possibly different fossilized taxa^14^.

Taking advantage of advanced spectroscopic tools (including synchrotron-based techniques), a number of studies have demonstrated that organic molecules may undergo only partial degradation during diagenesis in natural settings^15–24^. In parallel, laboratory experiments have helped evaluating the influence of key factors such as the pressure-temperature conditions, the redox conditions, the presence or absence of a fluid or of certain mineral phases on the degradation of organics^22, 25–35^. Altogether, these studies highlighted that burial-induced thermal degradation of organic molecules can be more abstruse than generally believed.

Here, we report results of thermal maturation experiments performed on two strains of unicellular microorganisms (the prokaryotic cyanobacteria *Gloeobacter violaceus* and the eukaryotic microalgae *Euglena gracilis*) and on two nitrogen-rich organic aerosols (PAMPRE and Nebulotron – see Methods) for different durations (1 to 100 days). The morphological and chemical changes of experimental organic residues have been characterized using scanning electron microscopy (SEM) and X-ray absorption near edge structure (XANES) spectroscopy, a synchrotron-based technique offering a precise estimation of the nitrogen-to-carbon (N/C) atomic ratio of organics^36, 37^ as well as key information about chemical structures, i.e. carbon and nitrogen speciation, with a submicrometric spatial resolution^24, 38^.

## Results

### Evolution of morphologies

**Figure 1:**
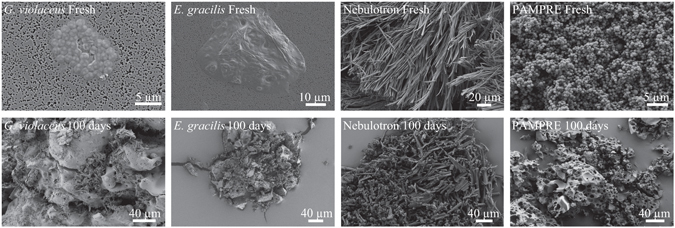
Evolution of fine-scale morphologies. SEM images of the organic and the corresponding residues of 100 day long advanced diagenesis experiments performed at 250 °C and 250 bars. Note that the morphology of Nebulotron has only slightly been degraded during the experiments.

SEM has been used to document morphological changes of the organic materials. Fresh *G*. *violaceus* exhibit spherical cells of about 1 μm in diameter, while fresh *E*. *gracilis* cells are approximately 30 μm in length and 10 μm in width (Fig. 1). Nebulotron are rod shaped particles of tens to hundreds of microns in length and approximately 2 µm in width. PAMPRE are spherical particles of about 0.5 µm in diameter. During diagenesis experiments, *G*. *violaceus*, *E*. *gracilis* and PAMPRE have evolved towards more or less oily residues with no particular morphologies while the initial morphology of Nebulotron has been partially preserved, even after 100 days (Fig. 1). Note the sponge-like texture of PAMPRE residues, likely resulting from gaseous compounds generation during the experiments.

### Evolution of N/C values

**Figure 2:**
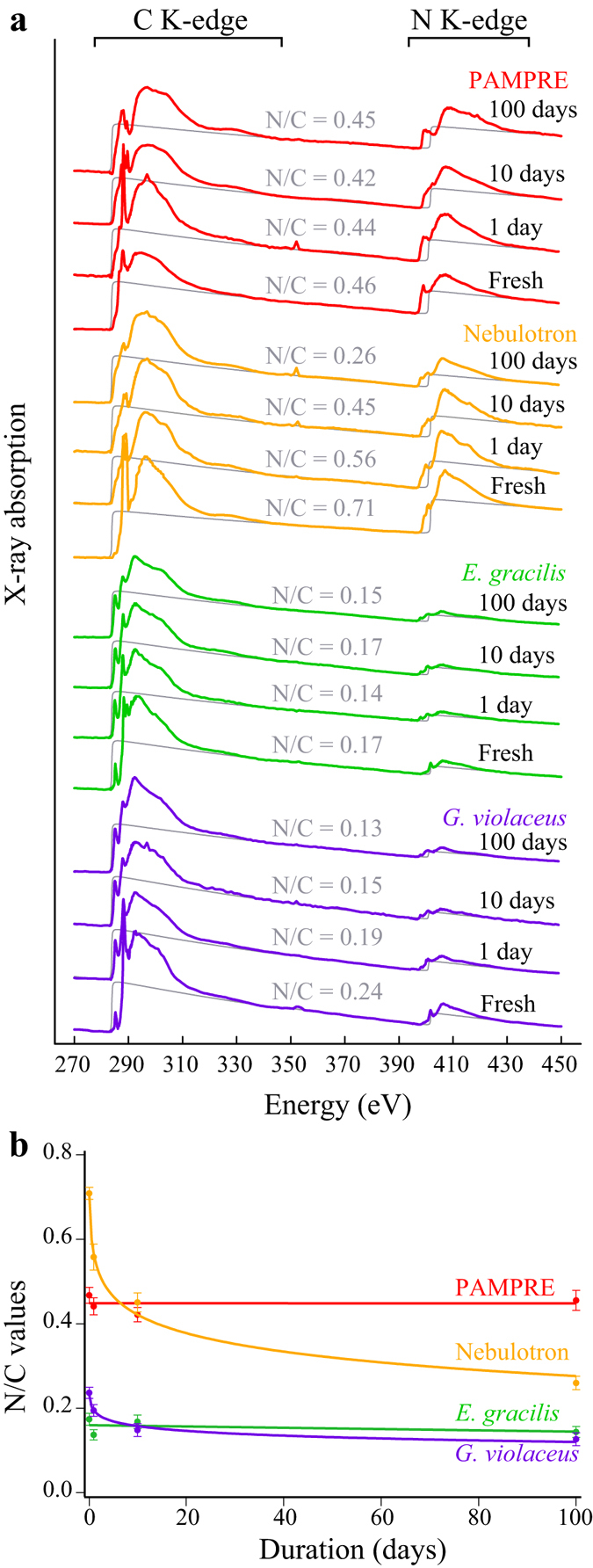
Evolution of N/C values. (**a**) X-ray absorption spectra of organic materials and the corresponding residues of 1, 10 and 100 day long advanced diagenesis experiments performed at 250 °C and 250 bars. (**b**) Evolution of the N/C values with experimental duration. Note that, depending on the organic precursor, the N/C ratio either decreases logarithmically (Nebulotron and *G*. *violaceus*) of remains nearly constant (PAMPRE and *E*. *gracilis*) with experimental duration.

Following Alleon *et al*.^37^, N/C values of the starting materials and experimental residues have been estimated from their X-ray absorption spectra (Fig. 2). N/C values of 0.24 and 0.17 (±0.01) have been determined for *G*. *violaceus* and *E*. *gracilis*, respectively. Nebulotron and PAMPRE aerosols exhibit higher nitrogen contents, with N/C values of 0.71 ± 0.01 and 0.46 ± 0.02, respectively, in good agreement with bulk measurements^39^. While the N/C values of *G*. *violaceus* and Nebulotron have significantly decreased during the experiments following a log-linear relationship with experimental duration, *E*. *gracilis* and PAMPRE residues exhibit N/C values quite similar to those of the starting materials, even after 100 days at 250 °C and 250 bars (Fig. 2).

### Evolution of chemical structures

#### Chemical structures of starting materials

**Figure 3:**
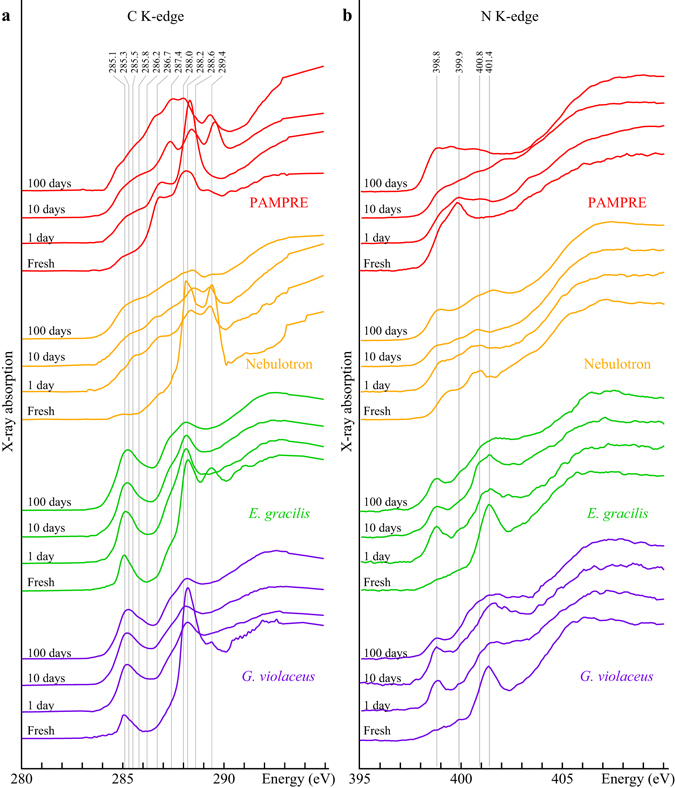
Evolution of XANES signatures. C-XANES (**a**) and N-XANES (**b**) spectra of organic materials and the corresponding residues of the 1, 10 and 100 day long advanced diagenesis experiments performed at 250 °C and 250 bars. Attribution of absorption features: 285.1 eV: aromatics/olefinics; 285.8–287.4 eV: imines/nitriles/carbonyls/phenols; 287.5–288.0 eV: aliphatics; 288.2 eV: amides; 288.6 eV: carboxyls/esters/acetals; 289.4 eV: hydroxyls; 398.8–399.9 eV: imines/nitriles/pyridines; 400.8/401.4 eV: amides.

Fresh *G*. *violaceus* exhibit a C-XANES spectrum typical of bacteria (Fig. 3a), with a main peak at 288.2 eV, assigned to the 1 s → π* electronic transitions in amide groups ((R_1_, R_2_)N–C=O), a peak at 289.4 eV corresponding to 1 s → 3p/σ* transitions in hydroxyl groups (C-OH), a peak at 285.1 eV, attributed to 1 s → π* transitions in aromatic or olefinic groups (C=C), and a large shoulder centered at about 287.4 eV, attributed to both 1 s → π* transitions (287.0–287.3 eV) in carbonyl (C=O) and phenolic (Ar-OH) groups and 1 s → 3p/σ* transitions (287.5–288.0 eV) in aliphatic (-C_x_H_y_) groups^19, 29, 40^. The C-XANES spectrum of fresh *E*. *gracilis* appears quite similar with a more intense peak at 289.4 eV (Fig. 3a), indicating a higher content of carbohydrates, likely stored inside the cells as starch granules and paramylon^41^. Fresh Nebulotron particles also display a C-XANES spectrum dominated by three absorption features at 285.1, 288.2 and 289.4 eV which respective intensities indicate a higher concentration of amide ((R_1_, R_2_)N–C=O) and hydroxyl groups (C-OH) and a lower concentration of aromatic or olefinic carbons (C=C) compared to *G*. *violaceus* and *E*. *gracilis*. The shoulder centered around 286.7 eV can be attributed to 1 s → π* electronic transitions in imine (C=N), nitrile (C≡N), carbonyl (C=O) and/or phenolic (Ar-OH) groups^42–44^. The C-XANES spectrum of fresh PAMPRE exhibits a different pattern with a broad peak centered around 288.2 eV, attributed to the contribution of 1 s → π* electronic transitions in amide groups ((R1, R2)N–C=O) and to that of 1 s → 3p/σ* transitions in various aliphatic groups mostly connected to nitrogen, and two shoulders, an intense one centered at about 286.7 eV and attributed to 1 s → π* electronic transitions in nitrile (C≡N) and a more gentle one centered at about 285.1 eV and attributed to 1 s → π* transitions in aromatic or olefinic groups (C=C)^42–44^.

Due to overlapping energies of several resonances, absorption peaks at the N *K*-edge cannot be univocally assigned to given functional groups^42, 45, 46^. Still, the N local coordination can be partly inferred based on the energy of absorption features^42, 45, 46^: imine, nitrile and pyridinic N will generate absorption features below 400 eV, while absorption features above 400 eV will indicate the presence of amide, nitro and pyrrolic N. Fresh *G*. *violaceus* and *E*. *gracilis* exhibit very similar N-XANES spectra with a main peak at 401.4 eV, likely related to amide groups that form the peptide bonds in proteins (the main N storage in microorganisms) and a wide absorption feature of low intensity in the range 398.8–399.9 eV, attributed to 1 s → π* transitions in either imine, nitrile or pyridine groups (Fig. 3b). The N-XANES spectrum of fresh Nebulotron particles is quite different: the absorption peak attributed to the contribution of 1 s → π* transitions in amide groups occurs at 400.8 eV instead of 401.4 eV, and a wide absorption peak, attributed to 1 s → π* transitions in either imine, nitrile or pyridine groups¸ can be observed at about 399.5 eV (Fig. 3b). The N-XANES spectrum of fresh PAMPRE does not display any absorption features above 400 eV, but a quite intense peak at 399,9 eV, attributed to the contribution of 1 s → π* transitions in nitrile groups, and a shoulder at about 398.8 eV, attributed to 1 s → π* transitions in imine groups^39^.

#### Chemical structures of experimental residues

The four organic materials have undergone a significant molecular evolution during the experiments: the relative intensities of C- and N-XANES peaks vary as a function of experimental duration (Fig. 3). Qualitatively, *G*. *violaceus* and *E*. *gracilis* have evolved in a similar manner. Their relative concentrations of amide and hydroxyl groups have decreased (as evidenced by the intensity decrease of the absorption peaks at 288.2/401.4 eV and 289.4 eV) while the relative concentrations of aromatic/olefinic groups and imine/nitrile/carbonyl/phenolic groups have increased (as evidenced by the intensity increase of the absorption features at 285.1/398.8–399.9 eV and 287.4/398.8 eV). Yet, while the molecular degradation of *G*. *violaceus* seems almost complete after 1 day, *E*. *gracilis* has evolved more progressively. With increasing experimental duration, both strains show a progressive shift of the aromatic/olefinic carbons absorption (from 285.1 to 285.3 eV). A similar progressive evolution is observed for Nebulotron (Fig. 3), except that Nebulotron residues have become enriched in imine/nitrile/carbonyl/phenolic groups (higher intensities of absorption in the ranges 285.8–287.4 eV and 398.8–399.9 eV). This is also the case for PAMPRE residues, despite a more chaotic chemical evolution (Fig. 3): carboxylic groups have formed within the first day (intense absorption peak at 288.4 eV), hydroxyl groups have appeared between 1 and 10 days (intense absorption peak at 289.5 eV) and aliphatic groups have become predominant between 10 and 100 days (absorption features in the 287.4–288.0 eV energy range).

## Discussion

SEM observations and XANES data show that the four organic materials do not degrade similarly when submitted to 250 °C and 250 bars, and remain distinct, even after 100 days, in terms of both morphology and molecular composition. Quite illustrative are the evolutions of the N/C values (Fig. 2). The present study evidences the different molecular transformations that chemically different organic materials may undergo when experimentally submitted to pressure and temperature conditions typical of burial-induced thermal diagenesis, thereby pointing out that organic molecular heterogeneities can withstand diagenesis. Of note, no simple correlation can be drawn between the evolutions of N/C, AI and UI values and the initial N/C values of the four organic materials, nor their initial morphologies or chemical structures, nor their biological or abiogenic nature. These observations illustrate both the diversity and the complexity of the degradation pathways that organic materials are likely to follow.

The N/C values of *G*. *violaceus* and Nebulotron have decreased with increasing experimental duration following apparent log-linear kinetic behaviors while those of *E*. *gracilis* and PAMPRE have remained nearly constant. Although Nebulotron initially exhibit N/C values 50% higher than those of PAMPRE, this is the opposite after 100 days. Similar trends can be evidenced for *G*. *violaceus* and *E*. *gracilis* despite the quite comparable initial chemical compositions of these microorganisms. Their chemical evolutions (Fig. 3) are typical of microorganisms having undergone thermal maturation^25, 29, 35^, i.e. a loss of heteroatomic functional groups concomitant to an increase of the relative abundance of aromatic structures. Yet, their nitrogen speciation has evolved slightly differently even though these microorganisms could not initially be distinguished based on their N-XANES spectra (Fig. 3).

**Figure 4:**
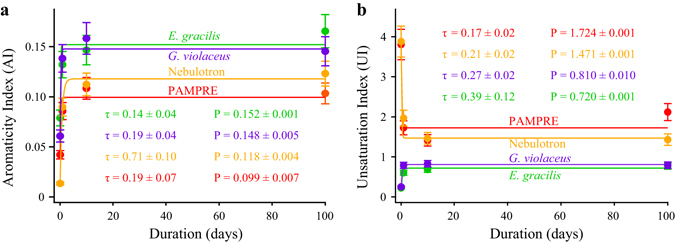
Evolution of XANES spectral parameters. AI (**a**) and UI (**b**) values of organic materials and the corresponding residues of 1, 10 and 100 day long advanced diagenesis experiments performed at 250 °C and 250 bars. Note that the evolutions of both AI and UI with experimental duration are well described by a first order kinetic law for the four organic materials (colored curves).

To discuss the chemical evolution of the starting organic materials in a more quantitative manner, deconvolution of C-XANES spectra has been performed, thereby allowing the extraction of two parameters: the aromaticity index (AI) and the unsaturation index (UI). The AI values of the four organic materials drastically increase during the first days and then reach a plateau value (in less than 10 days) that is higher for *G*. *violaceus* and *E*. *gracilis* than for Nebulotron and PAMPRE (Fig. 4a). The UI values of the four organic materials reveal a different evolution trend (Fig. 4b). With increasing experimental duration, the UI values of both *G*. *violaceus* and *E*. *gracilis* drastically increase during the very first days and then reach a plateau value that remains lower than 1. In contrast, the UI values of PAMPRE and Nebulotron drastically decrease during the very first days and then reach a plateau value that remains higher than 1. These two opposite trends indicate that, while the thermal maturity increases for all samples as indicated by the AI values, the concentration of C=N, C≡N, C=O and Ar-OH groups remains higher than that of aromatic groups in residues of Nebulotron and PAMPRE (UI > 1) but lower than that of aromatic groups in residues of *E*. *gracilis* and *G*. *violaceus* (UI < 1).

The evolutions of AI and UI values of the four organic materials with experimental duration are well modelled by a first-order kinetic law according to:

$${({\rm{AI}},{\rm{UI}})}_{{\rm{t}}}={\rm{P}}{(1-\exp (-{\rm{t}}/{\rm{\tau }}))}^{{\rm{\alpha }}},$$

with t the experimental duration, P the plateau value and τ the characteristic time for the evolutions of either AI or UI values. The parameter α accounts for the increasing (α = 1) or decreasing (α = −1) evolution of AI and UI values with experimental duration. For both AI and UI values, while the calculated τ values are significantly lower than 1 for the four organic materials, the obtained P values differ from one organic material to another. For instance, the P values of *G*. *violaceus* (P_AI_ = 0.148 ± 0.005; P_UI_ = 0.810 ± 0.010) and *E*. *gracilis* (P_AI_ = 0.152 ± 0.001; P_UI_ = 0.720 ± 0.001) significantly differ from the ones of Nebulotron (P_AI_ = 0.118 ± 0.004; P_UI_ = 1.471 ± 0.001) and PAMPRE (P_AI_ = 0.099 ± 0.007; P_UI_ = 1.724 ± 0.001).

Here, the four organic materials have undergone degradation only during the very first days of experiments. Even the AI and UI values of PAMPRE reach a plateau within a day despite the more chaotic evolution of these aerosols during thermal maturation. The low τ values obtained from the simple modelling of the evolutions of the AI and UI values highlight that a metastable thermodynamic equilibrium, likely consisting of a mixture of gas, oil and a mature solid residue^47, 48^, has been achieved during the very first days of experimental diagenesis (99% of the plateau (5τ) is reached in 3.5 days for Nebulotron and within the first day for *G*. *violaceus*, *E*. *gracilis* and PAMPRE). Basic extrapolations of the obtained kinetic laws for much longer durations suggest that no additional degradation would occur during geological times. In other words, the present results suggest that initially chemically different organic materials can remain different after several million years of diagenesis.

Advanced chemical characterization using spatially resolved spectroscopy techniques have been tentatively used to discuss the biological affinities of organic-walled microfossils^16, 24, 49–53^. Still, most of the witnesses of the first steps of the history of Life are unicellular organic-walled microfossils of unknown and probably varied biological affinities which fall into the category of acritarchs^2, 54, 55^. As illustrated here, consistently with reports of partly preserved biogenic organic molecules in the fossil record^15–24^, thermal maturation does not necessarily result in a convergence in the composition of organic materials from different sources. Thus, the present results suggest that enigmatic fossil acritarchs of different biological affinities could be distinguished based on their molecular compositions provided that they underwent a similar diagenetic history.

As for all laboratory simulations, the present experiments do not exactly mimic what really occurs in natural settings during millions of years^22, 25, 29, 31, 33–35^. Biodegradation and early taphonomic processes that may occur within sediments have not been simulated here, even though they may also alter the chemical composition of organic materials before the beginning of thermal maturation^14, 56^. Organic maturation processes in natural settings may also be strongly affected by the circulation of fluids^57–60^ or by the presence of certain mineral phases^28, 29, 35, 61–64^, which respective influence still needs to be rigorously investigated. In any case, besides its potential applications for the generation of thermostable biocomposites following the extreme biomimetic concepts^65, 66^, the present study highlights the pertinence of performing laboratory experiments to strengthen the current mechanistic understanding of organic material degradation processes, and thence, to accurately reconstruct the original chemical composition of ancient organic microfossils.

## Materials and Methods

### Selected organic materials

Two strains of unicellular oxygenic photosynthetic microorganisms have been selected: the prokaryotic cyanobacteria *Gloeobacter violaceus* (PCC 7421) and the eukaryotic microalgae *Euglena gracilis* (n°1224-5d - Cambridge). Both strains have been cultured at ambient temperature at IMPMC (Paris, France), *G*. *violaceus* in a regular BG-11 culture medium as done by Couradeau *et al*.^67^, and *E*. *gracilis* in a culture medium composed of KH_2_PO_4_ (0.5 g·L^−1^), MgSO_4_, 7H_2_O (0.5 g·L^−1^), CaCl_2_, 2H_2_O (0.26 g·L^−1^), (NH_4_)_2_HPO_4_ (0.5 g·L^−1^), a complement of vitamins, zinc (as ZnSO_4_), iron (as FeCl_3_), and manganese (as MnSO_4_) as done by Miot *et al*.^68, 69^. Cells of both strains were rinsed 3 times in bi-distilled water and dried in an oven at 50 °C under vacuum for 3 days prior to the experiments.

Two synthetic organic areosols (called Nebulotron and PAMPRE hereafter) have been selected for the present experimental study. These solid organics have been synthesized at CRPG (Nancy, France) by plasma discharge in gaseous mixtures using two different experimental setups^39^. Nebulotron are nitrogen and oxygen-rich aerosols condensed from a gaseous mixture made of 80% N_2_ and 20% CO while PAMPRE are nitrogen and hydrogen-rich aerosols condensed from a gaseous mixture made of 95% N_2_ and 5% CH_4_.

### Thermal maturation experiments

About 1 mg of precursors have been placed into individual gold capsules that have then been sealed under argon atmosphere using an electrical arc and placed in Parr^©^ autoclaves in which temperature and pressure conditions typical of burial-induced diagenesis (250 ± 2.5 °C and 250 ± 2.5 bars) have been maintained for 1, 10 and 100 days, thereby allowing kinetic investigations. All experiments have been repeated three times. Experimental solid residues have been recovered from the capsules and stored at 4 °C before being prepared for microscopic and spectroscopic analyses.

### Scanning Electron Microscopy (SEM)

SEM observations have been performed on pieces of experimental residues deposited on aluminum stubs and coated with 15 nm of gold, using the SEM-FEG ultra 55 Zeiss (IMPMC - Paris, France) microscope operating at a 3 kV accelerating voltage and a working distance of 3 mm for secondary electrons analyses (SE2 detector).

### X-ray Absorption Near Edge Structure (XANES) spectroscopy

#### XANES data acquisition procedure

XANES data have been collected on the 10ID-1 beamline (SM beamline)^70^ at the Canadian Light Source (CLS). The CLS SM beamline uses a monochromated X-ray beam spanning the 130–2500 eV range. This beam is generated with an elliptically polarized undulator (EPU) inserted in the 2.9 GeV, 250–100 mA, CLS synchrotron storage ring. The microscope chamber is first pumped down to 100 mTorr after sample insertion and then back*-*filled with He gas. A 100 nm thick titanium filter is used to remove the contribution of second order light. Energy calibration is done using the well-resolved 3p Rydberg peak of gaseous CO_2_ at 294.96 eV for the C *K-*edge and using the 1 s → π* photoabsorption resonance of gaseous N_2_ at 400.8 eV for the N *K-*edge. Image stacks have been collected with energy increments of 0.1 eV over the 250–450 eV energy range with a dwell time of one millisecond or less per pixel to prevent irradiation damages as recommended by Wang *et al*.^71^. Data processing has been done using the aXis2000 software package^72^. The N/C atomic ratio has been determined following the procedure developed by Alleon *et al*.^37^. The C- and N-XANES spectra shown here were averaged among triplicate samples and correspond to homogeneous areas of several tens of squared micrometers.

#### C-XANES data deconvolution procedure

A deconvolution procedure in three steps has been applied to C-XANES spectra: (i) background subtraction, (ii) normalization to the total carbon content, and (iii) spectral fitting using Gaussian functions. Background subtraction classically consists in the subtraction of a linear regression over the 270–282 eV energy range. Then, following Barré *et al*.^73^, C-XANES spectra have been normalized to their area between 280 eV and 291.5 eV, thereby ensuring chemical consistency (a spectrum showing a more prominent absorption than others at a given energy must have a less intense absorption at the energy of the other functional groups). Gaussian functions with a constant full-width at half maximum (0.6 eV) have been used for signal deconvolution. Their positions have been fixed following Myneni^42^, Dhez *et al*.^74^ and Solomon *et al*.^75^: 284.4 eV, quinones; 285.0 & 285.4 eV, aromatics and/or olefinics; 285.8 eV, imines; 286.2, 286.6 & 287.1 eV, nitriles/carbonyls/phenols; 287.7 eV, aliphatics; 288.2 eV, amides; 288.6 eV, carboxylics; 289.1 eV, aldehydes; 289.4 eV, hydroxyls; 289.9 eV, aliphatics; 290.3 eV, carbonates. Following Bernard *et al*.^31, 76^, Le Guillou *et al*.^77, 78^ and Alleon *et al*.^35^, two semi-quantitative parameters have been extracted from the XANES spectra: (1) the ‘aromaticity index’ (AI) that corresponds to the contribution of aromatic/olefinic carbons (i.e. the sum of the areas of the three Gaussian functions used to deconvolve the absorption signal in the range 284.4–285.4 eV), and (2) the ‘unsaturation index’ (UI) that corresponds to the contribution of C≡N, C=N, C=O and Ar-OH (i.e. the sum of the areas of the three Gaussian functions used to deconvolve the absorption signal in the range 285.8–286.6 eV) normalized to the aromaticity index. The degree of uncertainty associated with these estimated values (±10%, 1 SD) is inherited from the normalization procedure.

## References

[1] Bernard S, Papineau D (2014). Graphitic carbons and biosignatures. Elements.

[2] Knoll AH (2014). Paleobiological perspectives on early eukaryotic evolution. Cold Spring Harb. Perspect. Biol..

[3] Briggs DE, Summons RE (2014). Ancient biomolecules: their origins, fossilization, and role in revealing the history of life. Bio. Essays.

[4] Bernard S, Horsfield B (2014). Thermal maturation of gas shale systems. Annu. Rev. Earth Planet. Sci..

[5] Beyssac O, Rumble D (2014). Graphitic carbon: a ubiquitous, diverse, and useful geomaterial. Elements.

[6] Buseck PR, Beyssac O (2014). From organic matter to graphite: Graphitization. Elements.

[7] Tegelaar EW, De Leeuw JW, Derenne S, Largeau C (1989). A reappraisal of kerogen formation. Geochim. Cosmochim. Acta.

[8] Eglinton G, Logan GA (1991). Molecular Preservation. Philos. Trans. R. Soc. Lond. B Biol. Sci..

[9] van Bergen P (1995). Resistant biomacromolecules in the fossil record. Acta Bot. Neerl..

[10] Briggs DEG (1999). Molecular taphonomy of animal and plant cuticles: selective preservation and diagenesis. Philos. Trans. R. Soc. Lond. B Biol. Sci..

[11] Derenne S, Largeau C (2001). A review of some important families of refractory macromolecules: composition, origin, and fate in soils and sediments. Soil Science.

[12] Stankiewicz BA (1998). Molecular taphonomy of arthropod and plant cuticles from the Carboniferous of North America: implications for the origin of kerogen. J. Geol. Soc..

[13] Gupta NS (2008). Molecular taphonomy of macrofossils from the Cretaceous Las Hoyas Formation, Spain. Cretaceous Res..

[14] Briggs DEG, McMahon S (2016). The role of experiments in investigating the taphonomy of exceptional preservation. Palaeontology.

[15] Bernard S (2007). Exceptional preservation of fossil plant spores in high-pressure metamorphic rocks. Earth Planet. Sci. Lett..

[16] Bernard S (2009). Ultrastructural and chemical study of modern and fossil sporoderms by Scanning Transmission X-ray Microscopy (STXM). Rev. Palaeobot. Palynol..

[17] Bernard S, Benzerara K, Beyssac O, Brown GE (2010). Multiscale characterization of pyritized plant tissues in blueschist facies metamorphic rocks. Geochim. Cosmochim. Acta.

[18] Boyce CK, Abrecht M, Zhou D, Gilbert PUPA (2010). X-ray photoelectron emission spectromicroscopic analysis of arborescent lycopsid cell wall composition and Carboniferous coal ball preservation. Int. J. Coal. Geol..

[19] Cody GD (2011). Molecular signature of chitin-protein complex in Paleozoic arthropods. Geology.

[20] Cosmidis J (2013). Nanometer‐scale characterization of exceptionally preserved bacterial fossils in Paleocene phosphorites from Ouled Abdoun (Morocco). Geobiology.

[21] Cosmidis J, Benzerara K, Menguy N, Arning E (2013). Microscopy evidence of bacterial microfossils in phosphorite crusts of the Peruvian shelf: Implications for phosphogenesis mechanisms. Chem. Geol..

[22] Ehrlich H (2013). Discovery of 505-million-year old chitin in the basal demosponge Vauxia gracilenta. Sci. Rep..

[23] Wysokowski M (2014). Identification of chitin in 200-million-year-old gastropod egg capsules. Paleobiology.

[24] Alleon J (2016). Molecular preservation of 1.88 Ga Gunflint organic microfossils as a function of temperature and mineralogy. Nat. Commun..

[25] Schiffbauer JD (2012). Thermally‐induced structural and chemical alteration of organic‐walled microfossils: an experimental approach to understanding fossil preservation in metasediments. Geobiology.

[26] McNamara ME (2013). The fossil record of insect color illuminated by maturation experiments. Geology.

[27] McNamara ME, Briggs DE, Orr PJ, Field DJ, Wang Z (2013). Experimental maturation of feathers: implications for reconstructions of fossil feather colour. Biol. Lett..

[28] Li J, Benzerara K, Bernard S, Beyssac O (2013). The link between biomineralization and fossilization of bacteria: Insights from field and experimental studies. Chem. Geol..

[29] Li J (2014). Impact of biomineralization on the preservation of microorganisms during fossilization: An experimental perspective. Earth Planet. Sci. Lett..

[30] Fraser WT (2014). Changes in spore chemistry and appearance with increasing maturity. Rev. Palaeobot. Palynol..

[31] Bernard S, Benzerara K, Beyssac O, Balan E, Brown GE (2015). Evolution of the macromolecular structure of sporopollenin during thermal degradation. Heliyon.

[32] Colleary C (2015). Chemical, experimental, and morphological evidence for diagenetically altered melanin in exceptionally preserved fossils. Proc. Natl. Acad. Sci..

[33] Picard A, Kappler A, Schmid G, Quaroni L, Obst M (2015). Experimental diagenesis of organo-mineral structures formed by microaerophilic Fe (II)-oxidizing bacteria. Nat. commun..

[34] Picard A, Obst M, Schmid G, Zeitvogel F, Kappler A (2015). Limited influence of Si on the preservation of Fe mineral‐encrusted microbial cells during experimental diagenesis. Geobiology.

[35] Alleon J (2016). Early entombment within silica minimizes the molecular degradation of microorganisms during advanced diagenesis. Chem. Geol..

[36] Cody GD (2008). Quantitative organic and light‐element analysis of comet 81P/Wild 2 particles using C‐, N‐, and O‐μ‐XANES. Meteorit. Planet. Sci..

[37] Alleon J, Bernard S, Remusat L, Robert F (2015). Estimation of nitrogen-to-carbon ratios of organics and carbon materials at the submicrometer scale. Carbon.

[38] Gueriau P, Bernard S, Bertrand L (2016). Advanced synchrotron characterization of paleontological specimens. Elements.

[39] Kuga M (2014). Nitrogen isotopic fractionation during abiotic synthesis of organic solid particles. Earth Planet. Sci. Lett..

[40] Robin N (2015). Calcification and Diagenesis of Bacterial Colonies. Minerals.

[41] Guttman HN (1971). Internal cellular details of Euglena gracilis visualized by scanning electron microscopy. Science.

[42] Myneni SCB (2002). Soft X-ray spectroscopy and spectromicroscopy studies of organic molecules in the environment. Rev. Mineral. Geochem..

[43] Shard AG (2004). A NEXAFS examination of unsaturation in plasma polymers of allylamine and propylamine. J. Phys. Chem. B.

[44] Nuevo M (2011). XANES analysis of organic residues produced from the UV irradiation of astrophysical ice analogs. Adv. Space Res..

[45] Leinweber P (2007). Nitrogen K-edge XANES – an overview of reference compounds used to identify unknown organic nitrogen in environmental samples. J. Synchrotron Radiat..

[46] Kiersch K, Kruse J, Regier TZ, Leinweber P (2012). Temperature resolved alteration of soil organic matter composition during laboratory heating as revealed by C and N XANES spectroscopy and Py-FIMS. Thermochim. Acta.

[47] Helgeson HC, Owens CE, Knox AM, Richard L (1998). Calculation of the standard molal thermodynamic properties of crystalline, liquid, and gas organic molecules at high temperatures and pressures. Geochim. Cosmochim. Acta.

[48] Helgeson HC, Richard L, McKenzie WF, Norton DL, Schmitt A (2009). A chemical and thermodynamic model of oil generation in hydrocarbon source rocks. Geochim. Cosmochim. Acta.

[49] Javaux EJ, Marshal CP (2006). A new approach in deciphering early protist paleobiology and evolution: Combined microscopy and microchemistry of single Proterozoic acritarchs. Rev. Palaeobot. Palynol..

[50] Igisu M (2009). Micro-FTIR spectroscopic signatures of Bacterial lipids in Proterozoic microfossils. Precambrian Res..

[51] Dhamelincourt MC (2010). Laser Raman micro-spectroscopy of Proterozoic and Palaeozoic organic-walled microfossils (acritarchs and prasinophytes) from the Ghadamis Basin, Libya and Volta Basin, Ghana. J. Spectro.

[52] Dutta S, Hartkopf-Fröder C, Witte K, Brocke R, Mann U (2013). (2013). Molecular characterization of fossil palynomorphs by transmission micro-FTIR spectroscopy: Implications for hydrocarbon source evaluation. Int. J. Coal Geol..

[53] Bobroff V, Chen HH, Javerzat S, Petibois C (2016). What can infrared spectroscopy do for characterizing organic remnant in fossils?. Trends Anal. Chem..

[54] Evitt WR (1963). A discussion and proposals concerning fossil dinoflagellates, hystrichospheres, and acritarchs, I. Proc. Natl. Acad. Sci.

[55] Servais T (1996). Some considerations on acritarch classification. Rev. Palaeobot. Palynol..

[56] Iniesto M (2015). Preservation in microbial mats: mineralization by a talc-like phase of a fish embedded in a microbial sarcophagus. Front. Earth Sci.

[57] Lewan, M. D. Laboratory simulation of petroleum formation. In *Organic Geochemistry* (pp. 419–442). Springer US. ISBN: 978-1-4613-6252-4, doi:10.1007/978-1-4615-2890-6_18 (1993).

[58] Seewald JS (2001). Aqueous geochemistry of low molecular weight hydrocarbons at elevated temperatures and pressures: Constraints from mineral buffered laboratory experiments. Geochim. Cosmochim. Acta.

[59] Boudou JP (2008). Organic nitrogen chemistry during low-grade metamorphism. Geochim. Cosmochim. Acta.

[60] Lewan MD, Roy S (2011). Role of water in hydrocarbon generation from Type-I kerogen in Mahogany oil shale of the Green River Formation. Org. Geochem..

[61] Seewald JS, Eglinton LB, Ong YL (2000). An experimental study of organic-inorganic interactions during vitrinite maturation. Geochim. Cosmochim. Acta.

[62] Briggs DE (2003). The role of decay and mineralization in the preservation of soft-bodied fossils. Annu. Rev. Earth Planet. Sci..

[63] Anderson EP, Schiffbauer JD, Xiao S (2011). Taphonomic study of Ediacaran organic-walled fossils confirms the importance of clay minerals and pyrite in Burgess Shale–type preservation. Geology.

[64] Schiffbauer JD (2014). A unifying model for Neoproterozoic–Palaeozoic exceptional fossil preservation through pyritization and carbonaceous compression. Nat. Commun..

[65] Wysokowski M (2015). Poriferan chitin as a versatile template for extreme biomimetics. Polymers.

[66] Ehrlich, H. (editor). Extreme Biomimetics. Springer International Publishing. ISBN: 978-3-319-45338-5, doi:10.1007/978-3-319-45340-8 (2017).

[67] Couradeau E (2012). An early-branching microbialite cyanobacterium forms intracellular carbonates. Science.

[68] Miot J (2008). XAS study of arsenic coordination in Euglena gracilis exposed to arsenite. Environ. Sci. Technol..

[69] Miot J (2009). Speciation of arsenic in Euglena gracilis cells exposed to As (V). Environ. Sci. Technol..

[70] Kaznatcheev KV (2007). Soft X-ray spectromicroscopy beamline at the CLS: commissioning results. Nucl. Instr. Meth. Phys. Res. A.

[71] Wang J (2009). Radiation damage in soft X-ray microscopy. J. Electron Spectrosc. Relat. Phenom..

[72] Hitchcock, A. aXis 2000 – Analysis of X-ray Images and Spectra. http://unicorn.mcmaster.ca/aXis2000.html (2016).

[73] Barré P (2016). The energetic and chemical signatures of persistent soil organic matter. Biogeochemistry.

[74] Dhez O, Ade H, Urquhart SG (2003). Calibrated NEXAFS spectra of some common polymers. J. Electron Spectrosc. Relat. Phenom..

[75] Solomon D (2009). Carbon (1s) NEXAFS spectroscopy of biogeochemically relevant reference organic compounds. Soil Sci. Soc. Am. J.

[76] Bernard S (2012). Geochemical evolution of organic-rich shales with increasing maturity: A STXM and TEM study of the Posidonia Shale (Lower Toarcian, northern Germany). Mar. Pet. Geol..

[77] Le Guillou C, Remusat L, Bernard S, Brearley AJ, Leroux H (2013). Amorphization and D/H fractionation of kerogens during experimental electron irradiation: comparison with chondritic organic matter. Icarus.

[78] Le Guillou C, Bernard S, Brearley AJ, Remusat L (2014). Evolution of organic matter in Orgueil, Murchison and Renazzo during parent body aqueous alteration: *in situ* investigations. Geochim. Cosmochim. Acta.

